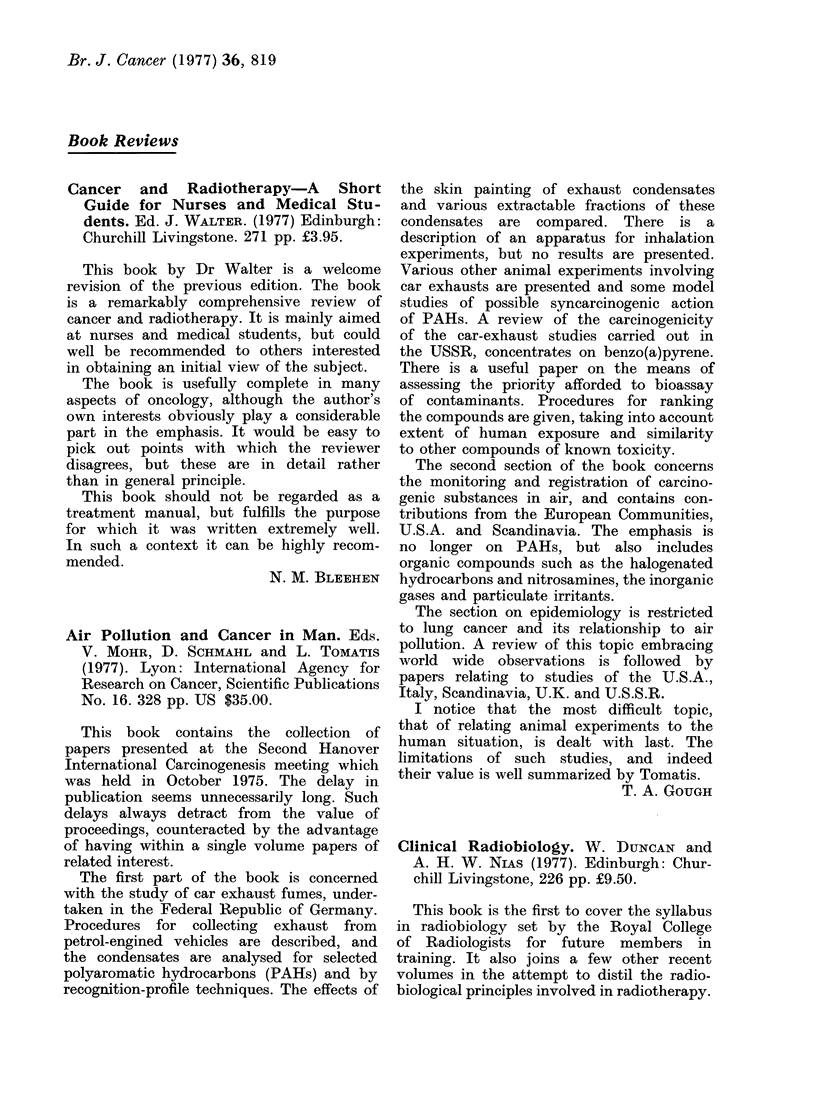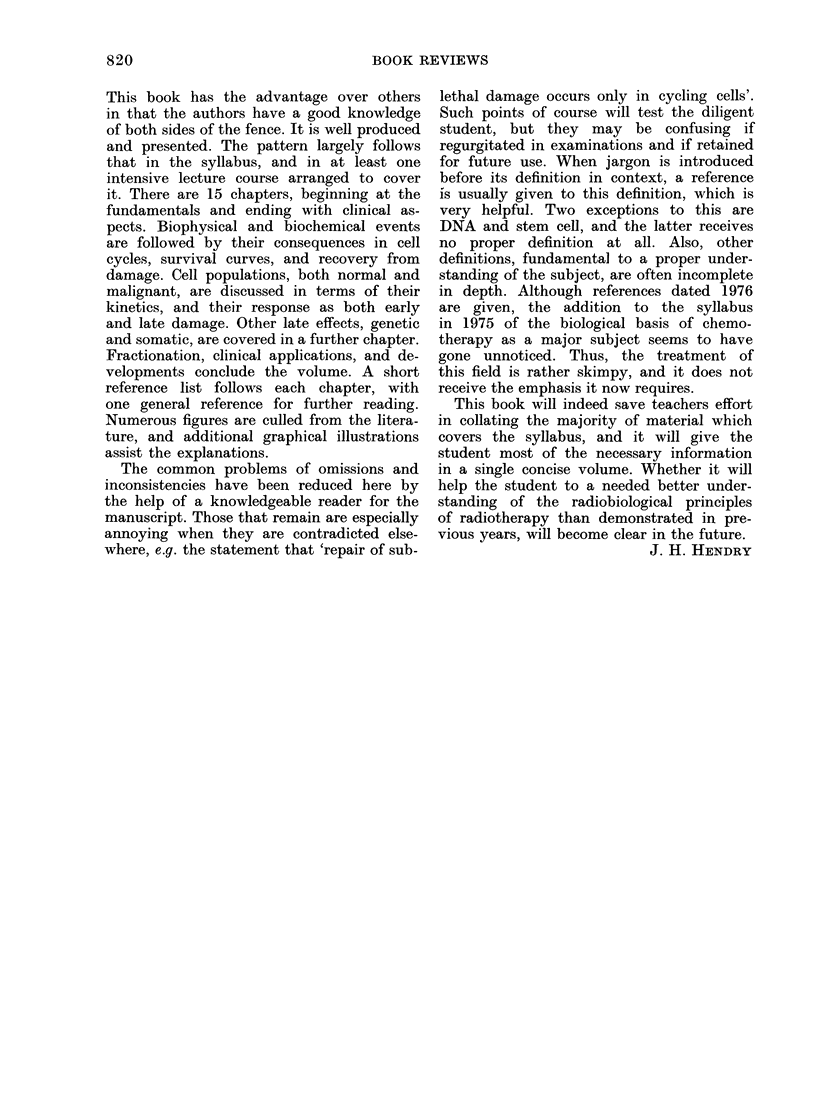# Clinical Radiobiology

**Published:** 1977-12

**Authors:** J. H. Hendry


**Clinical Radiobiology.** W. DUNCAN and A. H. W. NIAS (1977). Edinburgh: Churchill Livingstone, 226 pp. £9.50.

This book is the first to cover the syllabus in radiobiology set by the Royal College of Radiologists for future members in training. It also joins a few other recent volumes in the attempt to distil the radiobiological principles involved in radiotherapy.

This book has the advantage over others in that the authors have a good knowledge of both sides of the fence. It is well produced and presented. The pattern largely follows that in the syllabus, and in at least one intensive lecture course arranged to cover it. There are 15 chapters, beginning at the fundamentals and ending with clinical aspects. Biophysical and biochemical events are followed by their consequences in cell cycles, survival curves, and recovery from damage. Cell populations, both normal and malignant, are discussed in terms of their kinetics, and their response as both early and late damage. Other late effects, genetic and somatic, are covered in a further chapter. Fractionation, clinical applications, and developments conclude the volume. A short reference list follows each chapter, with one general reference for further reading. Numerous figures are culled from the literature, and additional graphical illustrations assist the explanations.

The common problems of omissions and inconsistencies have been reduced here by the help of a knowledgeable reader for the manuscript. Those that remain are especially annoying when they are contradicted elsewhere, *e.g.* the statement that 'repair of sublethal damage occurs only in cycling cells'. Such points of course will test the diligent student, but they may be confusing if regurgitated in examinations and if retained for future use. When jargon is introduced before its definition in context, a reference is usually given to this definition, which is very helpful. Two exceptions to this are DNA and stem cell, and the latter receives no proper definition at all. Also, other definitions, fundamental to a proper understanding of the subject, are often incomplete in depth. Although references dated 1976 are given, the addition to the syllabus in 1975 of the biological basis of chemotherapy as a major subject seems to have gone unnoticed. Thus, the treatment of this field is rather skimpy, and it does not receive the emphasis it now requires.

This book will indeed save teachers effort in collating the majority of material which covers the syllabus, and it will give the student most of the necessary information in a single concise volume. Whether it will help the student to a needed better understanding of the radiobiological principles of radiotherapy than demonstrated in previous years, will become clear in the future.

J. H. Hendry